# Adaptive and non-adaptive convergent evolution in feather reflectance of California Channel Islands songbirds

**DOI:** 10.1098/rspb.2023.1914

**Published:** 2023-11-15

**Authors:** Cody K. Porter, Faye G. Romero, Dean C. Adams, Rauri C. K. Bowie, Eric A. Riddell

**Affiliations:** ^1^ Department of Ecology, Evolution, and Organismal Biology, Iowa State University, Ames, IA 50011, USA; ^2^ Department of Biology, University of Rochester, Rochester, NY 14620, USA; ^3^ Museum of Vertebrate Zoology and Department of Integrative Biology, University of California Berkeley, Berkeley, CA 94720, USA; ^4^ Department of Biology, University of North Carolina – Chapel Hill, Chapel Hill, NC 27599, USA

**Keywords:** camouflage, Channel Islands, convergent evolution, island melanism, plumage, thermoregulation

## Abstract

Convergent evolution is widely regarded as a signature of adaptation. However, testing the adaptive consequences of convergent phenotypes is challenging, making it difficult to exclude non-adaptive explanations for convergence. Here, we combined feather reflectance spectra and phenotypic trajectory analyses with visual and thermoregulatory modelling to test the adaptive significance of dark plumage in songbirds of the California Channel Islands. By evolving dark dorsal plumage, island birds are generally less conspicuous to visual-hunting raptors in the island environment than mainland birds. Dark dorsal plumage also reduces the energetic demands associated with maintaining homeothermy in the cool island climate. We also found an unexpected pattern of convergence, wherein the most divergent island populations evolved greater reflectance of near-infrared radiation. However, our heat flux models indicate that elevated near-infrared reflectance is not adaptive. Analysis of feather microstructure suggests that mainland-island differences are related to coloration of feather barbs and barbules rather than their structure. Our results indicate that adaptive and non-adaptive mechanisms interact to drive plumage evolution in this system. This study sheds light on the mechanisms driving the association between dark colour and wet, cold environments across the tree of life, especially in island birds.

## 1. Introduction

A recurring theme in biology is the evolution of similar phenotypes in similar environments by independently evolving lineages, broadly referred to as convergent evolution [1]. Many biologists view convergent evolution as evidence of adaptation driven by similar selective pressures in similar environments [2]. However, different selective pressures can also generate convergence [2], as can several non-adaptive mechanisms [3,4]. Rigorously evaluating adaptive hypotheses for convergent evolution thus requires testing the effects of convergent phenotypes on ecologically relevant axes of performance across taxa [1].

One of the most widespread yet enigmatic examples of convergent evolution is the repeated occurrence of dark organismal coloration in wet and cold environments [5–8]. Dark coloration in wet, cold environments has independently evolved several times within birds, reptiles, mammals, amphibians, arthropods and fungi [9–12]. This phenomenon has been especially well-described in birds throughout the World's islands ([13–16]; hereafter referred to as ‘island melanism’), which are significantly wetter and cooler than nearby mainland environments [17]. A classic hypothesis is that dark coloration enhances camouflage in humid environments (the ‘camouflage hypothesis'), which are generally dark owing to high organic content of soils and greater vegetation/cloud cover associated with greater precipitation [18]. Despite its prominence in the literature, evidence bearing on this hypothesis is limited [10]. Moreover, this hypothesis does not consider the role of temperature in shaping coloration. While many studies have linked dark coloration in ectotherms and cold regions to thermoregulatory benefits (the ‘thermal melanism hypothesis’; [9]), this hypothesis is less often applied to endotherms. Yet laboratory studies have shown that coloration can affect endotherm energetics [19] and recent comparative studies have found an association between dark coloration and cold temperatures in some endotherms [5–8,20]. The adaptive significance, if any, of dark coloration in wet, cold environments is thus unclear.

**Figure 1.**
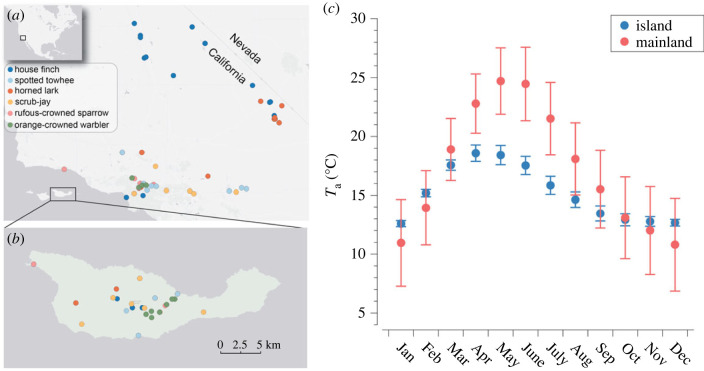
Sampling localities and their temperature. (*a*) We sampled museum specimens of six taxa from the mainland of southern California and (*b*) Santa Cruz Island. (*c*) Ambient temperature data from Worldclim (version 2.1) sampled from specimen sampling locations, showing that Santa Cruz Island is generally cooler than the mainland of southern California. Error bars in (*c*) are standard deviations. Museum sample sizes are as follows: scrub-jay (mainland = 33; island = 35), horned lark (mainland = 30; island = 19), house finch (mainland = 30; island = 15), orange-crowned warbler (mainland = 29; island = 20), spotted towhee (mainland = 30; island = 20), rufous-crowned sparrow (mainland = 10; island = 23).

Here, we combined reflectance spectrometry and phenotypic trajectory analyses with visual and heat flux simulations to test adaptive hypotheses for convergent evolution of island melanism in songbirds of the California Channel Islands. We focus on six species/population pairs on the mainland and the largest island in the archipelago, Santa Cruz Island (figure 1*a,b*). Despite occurring just 32 km off the coast of southern California, Santa Cruz Island is cooler than the mainland (figure 1*c*) owing to its proximity to the California Current [21]. Santa Cruz Island also has more fog and cloud cover [21], darker surface soils (average island soil albedo ± s.d. = 0.163 ± 0.034; average mainland soil albedo ± s.d. = 0.246 ± 0.082; https://websoilsurvey.nrcs.usda.gov/app), and more vegetation cover than the mainland (% island barren land = 0.009; % mainland barren land = 9.818; [22]). Consistent with broader patterns of animal coloration, 10 of 13 bird taxa endemic to the California Channel Islands have darker plumage than their mainland counterparts ([14]; electronic supplementary material, figure S1).

We studied multiple selective pressures driving convergence of feather coloration in populations of island birds. The camouflage hypothesis predicts convergence towards island melanism on dorsal feathers, which should reduce visual contrast between birds and the dark island environment under foggy skies and low ambient light [14,23]. Diurnal raptors, which appear to be the dominant predators of adult birds in the California Channel Islands [23], primarily attack prey from above [24], meaning dorsal surfaces are likely the primary target of selection for background matching (e.g. [20,25,26]). The thermal melanism hypothesis also predicts convergence towards island melanism on dorsal feathers because dorsal feathers are exposed to solar radiation but ventral feathers are largely shielded by the body. This hypothesis further predicts that elevated absorption of solar radiation by dark plumage should generate biologically significant reductions in thermoregulatory heating costs in cool environments [9]. Alternatively, dark plumage could be maladaptive if it increases solar absorptance and overheating. This could result in conflicting selective pressures if, for example, island melanism is favoured under the camouflage hypothesis. Such conflicting selective pressures could be resolved through a combination of dark visible plumage and high near-infrared feather reflectance, given that near-infrared radiation is invisible to animals and elevated near-infrared reflectance reduces heat gain from solar radiation [27]. We tested for a scenario of compensatory adaptation which predicts that the darkest island populations should evolve high near-infrared reflectance, thus reducing thermoregulatory demands associated with overheating (e.g. high evaporative water loss). Finally, we tested whether variation in feather microstructure can account for the mainland-island differences in visible and near-infrared feather reflectance we found.

## 2. Material and methods

### (a) Model parameterization from museum specimens

We parameterized visual and heat flux models by measuring biophysical characteristics of bird specimens in the Museum of Vertebrate Zoology at the University of California, Berkeley. The six songbird taxa studied here (California/island scrub-jay (*Aphelocoma californica/insularis*)*,* rufous-crowned sparrow (*Aimophila ruficeps*), horned lark (*Eremophila alpestris*)*,* house finch (*Haemorhous mexicanus*), orange-crowned warbler (*Leiothlypis celata*)*,* and spotted towhee (*Pipilo maculatus*)) were chosen based on the availability of specimens in the Museum of Vertebrate Zoology (see [28] for specimen information). A previous descriptive study [14] categorized five of these six taxa as having darker island forms, consistent with our reflectance measurements (electronic supplementary material, figure S2). The incidence of island melanism in our sample is thus similar to that of the total California Channel Islands bird community (10 out of 13 bird taxa; [14]). Nonetheless, our goal is not to test for the existence of island melanism, but rather to quantify the extent of known plumage convergence and test evolutionary mechanisms that may have caused it.

For each specimen, we estimated: (i) body shape; (ii) average feather length across the dorsum and ventrum; (iii) plumage depth across the dorsum and ventrum; and (iv) feather reflectance of the dorsum and ventrum. We assumed an average body shape, feather length, and plumage depth based upon allometric relationships between each of these traits and body mass developed from similar studies [29]. We obtained the mean body mass of each taxon from the VertNet database (http://www.vertnet.org/) based upon specimen collection points on Santa Cruz Island and mainland California (electronic supplementary material, appendix S1 Museum measurements for model parameterization). All available population genetic data suggest that mainland California is probably the ancestral source of bird taxa endemic to the California Channel Islands [30–36].

We measured dorsal and ventral feather reflectance every 1 nm from 350–2500 nm of each specimen using an ASD FieldSpec Pro spectroradiometer (ASD, Inc., 1625 S. Fordam Street, Suite 300, Longmont, CO 80503). Prior to each series of measurements on a specimen, we used a Spectralon white standard to standardize spectroradiometer readings. We measured feather reflectance using a tungsten halogen light source at a standardized distance (2 cm) from the feather surface with a 45° angle between the light source and fibre optic cable. The angle, distance, and area of measurement were standardized using a RPH-1 reflection probe holder (Ocean Optics, Inc., 8060 Bryan Dairy Road, Largo, FL 33777).

We measured reflectance from five feather patches on the dorsal and ventral surface of each specimen. A single measurement was taken from the crown or neck and four measurements spread across the breast or mantle using ViewSpec software (ASD, Inc.). We then estimated overall reflectance of the dorsal and ventral surfaces by averaging across dorsal and ventral feather patch measurements from each specimen. Finally, we corrected reflectance curves for solar radiation using the ASTM G-172 standard irradiance spectrum for dry air provided by SMARTs v. 2.9.2. We calculated the solar-corrected value by multiplying the intensity of solar radiation by the empirical reflectance, integrating across all wavelengths, and dividing by the total intensity of solar radiation [37]. Solar-correcting feather reflectance accounts for the uneven distribution of solar irradiance hitting the surface of Earth, thus providing a realistic estimate of feather reflectance in the field for the trajectory analyses and heat flux simulations described below.

### (b) Comparing patterns of feather reflectance

We quantified the direction and magnitude of mainland-island plumage divergence for each population pair using multivariate trajectory analyses in the R package RRPP (v. 0.4.2; [38]). For each population pair, plumage divergence can be described by a vector connecting the multivariate phenotypic centroid of mainland birds to the multivariate phenotypic centroid of island birds. The length of this vector describes the magnitude of mainland-island plumage divergence and its orientation describes the direction of divergence. The extent of convergent evolution can be quantitatively analysed by comparing the length and orientation of two vectors (similar vector lengths and orientations indicate convergence). The multivariate phenotypes in these analyses were matrices of solar-corrected feather reflectance every 1 nm across the ultraviolet (350–400 nm), visible (400–700 nm), near-infrared (700–2500 nm), or entire (350–2500 nm) portions of the light spectrum. We used the ‘pairwise’ function to compare vector lengths, vector orientations and phenotypic disparity of island and mainland populations. Greater disparity among taxa on the mainland than on the island is predicted if convergent evolution *sensu stricto* causes island melanism (e.g. [39]). We ran separate analyses for dorsal and ventral feather reflectance. In addition to the trajectory analyses, we also compared average feather reflectance of mainland and island populations every 100 nm from 350 to 2500 nm. The measure of feather reflectance divergence we used was: (reflectance of island birds − reflectance of mainland birds)/reflectance of island birds*100. Positive values indicate greater reflectance by island birds and negative values indicate greater reflectance by mainland birds.

To calculate the strength of phylogenetic signal in the evolution of ‘island melanism’, we collected categorical data on whether California Channel Island birds are darker than their mainland counterparts (based on Johnson [14], our own reflectance measurements, and those of Gamboa [40]). Given the generally high phylogenetic distances between species that have colonized the California Channel Islands, no single published phylogeny included all species. We therefore downloaded the ultrametric Tetrapoda supertree from Morrow *et al*. [41]. After pruning this tree to California Channel Islands taxa, we calculated the phylogenetic signal of a binary trait (presence or absence of island melanism) using the method of Fritz & Purvis [42] based on 1000 permutations. The phylogenetic tree and character states are depicted in the electronic supplementary material, figure S1.

### (c) Camouflage analyses

We quantified the discernability of birds with mainland and island plumage phenotypes against the background environment using the ‘coldist’ function in the R package pavo 2 [43]. This function applies the receptor-noise model of Vorobyev *et al*. [44] to calculate chromatic and achromatic contrast between objects (in this case, birds and their environment). The key parameters of this model are the raw reflectance spectra of birds and the background on which they typically reside, the spectral sensitives of a predator's photoreceptors, and the irradiance spectrum of light striking the birds and their background [44]. Reflectance spectra of birds were based on raw feather reflectance measurements from museum specimens (solar-corrected reflectance was not used in camouflage analyses because ‘coldist’ incorporates a user-defined irradiance spectrum). In terms of the environmental background that birds typically reside on, the taxa in our sample can be categorized as those that use sites with abundant barren land and exposed soil (horned lark, house finch, rufous-crowned sparrow) or those that use sites dominated by dense, green vegetation (scrub-jay, orange-crowned warbler, and spotted towhee; see the electronic supplementary material, table S11 for island and mainland habitat information). To reconstruct soil reflectance spectra for visual modelling, we first downloaded standard red, green, blue (sRGB) values of the nine most common Santa Cruz Island and California mainland surface soils from a soil colour dataset of the contiguous United States with a spatial resolution of 200 m [45]. These values were converted to reflectance curves using the ‘real colours’ approach of Burns [46]. Briefly, this method seeks the smoothest reflectance function corresponding to a point in sRGB colour space by solving the system of linear equations that minimize the square of the slope of the reflectance curve, summed over the entire curve ([46]; see the electronic supplementary material, appendix S1 Modelling camouflage, and figure S6 for reconstructed curves and comparison to empirical soil reflectance curves from other regions). For species using sites dominated by green vegetation, we incorporated the empirical reflectance spectra of oak (*Quercus* sp.) leaves [47]. Various oak species (namely, *Quercus* *agrifolia* and *Quercus* *pacifica*) are common components of the habitats used by scrub-jays, orange-crowned warblers, and spotted towhees (electronic supplementary material, table S11). The dominant predators of California Channel Islands songbirds appear to be several species of diurnal predatory birds [23], including *Accipiter cooperii*, *Accipiter* *striatus* and *Elanus leucurus* (members of Accipitridae), and *Falco columbarius*, *Falco* *peregrinus* and *Falco* *sparverius* (members of Falconidae; [48]). Therefore, we ran two separate ‘coldist’ analyses: one incorporating the violet-sensitive visual system of a peafowl (*Pavo cristatus*), which approximates that of Accipitridae [49], and the other incorporating the ultraviolet-sensitive visual system of a blue tit (*Cyanistes caeruleus*), which approximates that of Falconidae [49]. Finally, we modelled sky conditions on Santa Cruz Island and the California mainland to simulate irradiance conditions (see the electronic supplementary material, appendix S1 Modelling camouflage, for further details).

### (d) Heat flux simulations

We incorporated measurements of biophysical traits into a heat flux model to estimate the thermoregulatory demands of mainland and island birds on Santa Cruz Island. This model simulates heat balance using the morphological characteristics of each bird in a complex radiative and thermal environment. We incorporated feather reflectance into this model by converting solar reflectance (described above) to solar absorptance (1 − reflectance), which was then used to estimate the amount of direct, diffuse, and reflected solar radiation absorbed by the plumage. We also accounted for transmittance of solar radiation by modelling the probability of direct solar radiation passing through the plumage to the skin (see eqn 3 in [29] for more details). The simulation output produces estimates of net sensible heat flux (*Q*), which was calculated using an energy balance equation:

2.1 $$Q=\;M-E-C\frac{dT_{b}}{dt}=K_{e}(T_{b}-T_{e}),$$

where *M* is the heat generated through metabolic processes, *E* is the heat lost through evaporative water loss, *C* is the capacitance of the isothermal core, *T*_b_ is body temperature, *K*_e_ is the effective conductance and *T*_e_ is the operative temperature. Equation (2.1) estimates the heat flux required to maintain a stable body temperature given the morphology of the bird and its interaction with the environment. Net sensible heat flux is then used to estimate the required metabolic heat production or evaporative water loss necessary for homeothermy (electronic supplementary material, appendix S1 Heat flux simulation). In short, this model allows us to test whether mainland-island differences in feather reflectance significantly affect the thermoregulatory demands of birds in the cool island climate.

To test the adaptive significance of near-infrared feather reflectance, we artificially manipulated visible and near-infrared feather reflectance of the three darkest island populations (scrub-jay, horned lark and orange-crowned warbler) and tested the consequences for evaporative water loss. Specifically, we compared evaporative water loss rates of the island populations, which have low visible reflectance and high near-infrared reflectance (see Results) with artificial birds that had the observed low visible reflectance of island birds and low near-infrared reflectance of mainland birds (electronic supplementary material, appendix S1 Heat flux simulations). In effect, this analysis explored the thermoregulatory consequences of reducing the near-infrared reflectance of the darkest island birds without altering their ultraviolet or visible reflectance.

### (e) Feather microstructure and luminance

Certain aspects of feather microstructure (namely, barbule density) affect both visible and near-infrared reflectance [50] and could thus explain the mainland-island differences in feather reflectance we found (see Results). To test this hypothesis, we used a Keyence VHX-970F digital microscope to take standardized photographs of one dorsal and one ventral contour feather from the darkest island individual and the lightest mainland individual sampled for each taxon. Using ImageJ software, we measured barbule density as the number of barbules along a 1 mm barb transect in the middle of 10 barbs per feather. We also measured the unweighted luminance of 10 barbs per feather from a 0.01 mm × 0.2 mm section of each barb.

### (f) Statistics

**Figure 2.**
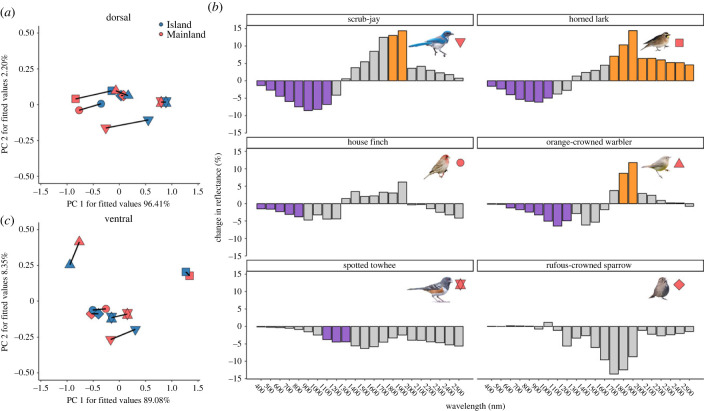
Dorsal (but not ventral) plumage reflectance has converged towards dark coloration in songbirds of Santa Cruz Island. (*a*) Multivariate trajectories of ultraviolet-visible dorsal plumage reflectance. Lines illustrate phenotypic trajectories from mainland (red) to island (blue) populations. Different symbols denote different species (next to images in panel (*b*)). The similar orientation of most phenotypic trajectories (except rufous-crowned sparrow) indicates convergent mainland-island plumage evolution. (*b*) Specifically, island birds' dorsal plumage has generally evolved less reflectance of ultraviolet-visible light (350–700 nm) and portions of near-infrared light (700–2500 nm) compared to mainland birds. Note that the most divergent island populations (scrub-jay, horned lark, and orange-crowned warbler) have also evolved greater reflectance of distant near-infrared light. The *y*-axis was calculated as: (reflectance of island birds − reflectance of mainland birds)/reflectance of island birds*100, such that positive values indicate greater reflectance by island birds and negative values indicate greater reflectance by mainland birds. Purple and orange 100 nm bins denote significantly greater reflectance by mainland or island birds, respectively. (*c*) The dissimilar orientation of visible ventral plumage trajectories indicates non-convergent mainland-island plumage evolution. High reflectance at all wavelengths loads negatively onto principal component (PC) 1 (electronic supplementary material, appendix), indicating that PC 1 describes achromatic variation, with high PC 1 values corresponding to darker birds. High reflectance values below 511 nm load negatively onto PC 2, while high reflectance values above 511 nm load positively onto PC 2 (electronic supplementary material, appendix). Therefore, PC 2 describes variation in the relative amount of short- to long-wavelength reflectance and high PC 2 values correspond to birds with greater long-wavelength reflectance.

**Figure 3.**
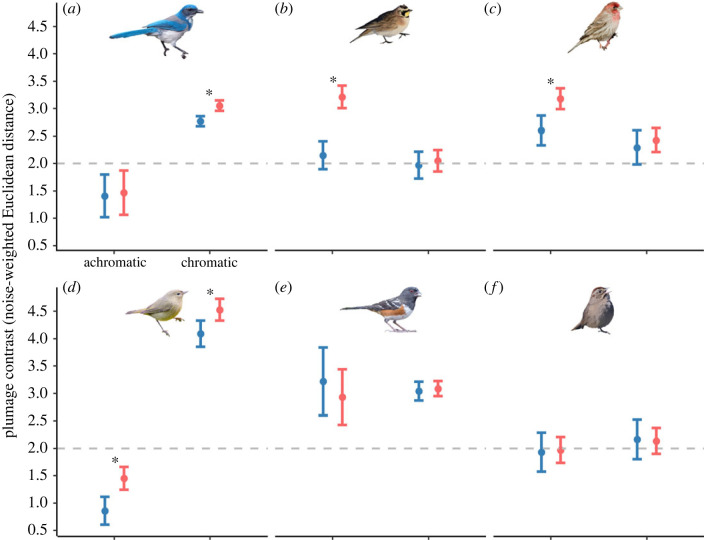
Island birds’ dark dorsal plumage enhances camouflage on Santa Cruz Island under a violet-sensitive visual model (see the electronic supplementary material, figure S5 for similar results under an ultraviolet-sensitive visual model). The *y*-axes depict achromatic and chromatic contrast between birds and their microhabitats on Santa Cruz Island in units of noise-weighted Euclidean distances. Blue values denote the visual contrast of island birds with the island environment and red values denote the visual contrast of mainland birds with the island environment. Island populations of (*a*) scrub-jays, (*b*) horned larks, (*c*) house finches, and (*d*) orange-crowned warblers show significantly less contrast with the island environment than their mainland counterparts (*p* ≤ 0.007 for all comparisons, denoted by asterisks). Island populations of (*e*) spotted towhees and (*f*) rufous-crowned sparrows did not differ from their mainland counterparts in contrast with the island environment (*p* > 0.675 for all comparisons). Error bars denote bootstrapped 95% confidence intervals (based on 2000 replicates). The dashed line at 2.0 indicates the threshold for avian visual discrimination assumed in most studies, though birds can detect values of 1 under some conditions [51].

Statistical analyses were conducted in R (v. 4.2.1) using two-sample *t*-tests with a Holm-Bonferroni correction for multiple comparisons (mainland-island reflectance differences depicted in figure 2*b*) and linear models (mainland-island differences in visual contrast with the environment depicted in figure 3).

## 3. Results

### (a) Convergent evolution of dorsal but not ventral plumage

For total dorsal plumage reflectance (350–2500 nm wavelengths), we found positive correlation coefficients (average ± s.e. = 0.285 ± 0.073) and acute angles (average ± s.e. = 71.094 ± 8.119°) between pairs of mainland-island trajectories, indicating similar directions of plumage evolution across taxa [51]. The extent of convergence was greater in the visible portion of the light spectrum (figure 2*a*; average correlation coefficient ± s.e. = 0.489 ± 0.181; average angle ± s.e. = 48.444 ± 13.217°), especially when rufous-crowned sparrow (the major outlier) was excluded (average correlation coefficient ± s.e. = 0.968 ± 0.020; average angle ± s.e. = 13.608 ± 3.514°). Disparity in visible reflectance between species on the island (average ± s.e. = 0.028 ± 0.005) was lower than on the mainland (average ± s.e. = 0.229 ± 0.051), pointing to convergent evolution *sensu stricto* in this system. Compared to their mainland counterparts, island populations have generally evolved reduced dorsal reflectance of visible and portions of near-infrared light (figure 2*b*). The most phenotypically divergent island populations (scrub-jay, horned lark, and orange-crowned warbler) also evolved greater dorsal reflectance of distant near-infrared light (figure 2*b*).

For total ventral plumage reflectance, we found negative pairwise correlation coefficients (average ± s.e. = −0.024 ± 0.162) and orthogonal angles (average ± s.e. = 92.308 ± 10.558°) between pairs of mainland-island trajectories, indicative of non-convergent evolution. Contrary to the dorsal plumage results, convergence in ventral plumage was lower in the visible portion of the light spectrum (figure 2*c*; average correlation coefficient ± s.e. = −0.139 ± 0.183; average angle ± s.e. = 100.412 ± 12.560°).

We also found substantial variation in the magnitude of mainland-island plumage divergence, with rufous-crowned sparrows and spotted towhees showing notably weak mainland-island divergence (figure 2; electronic supplementary material, tables S1–S8). Importantly, the occurrence of island melanism in the broader community of California Channel Islands birds is distributed randomly with respect to phylogeny (Fritz and Purvis' *D* = 1.003, *p* = 0.389; electronic supplementary material, figure S1), suggesting that intrinsic constraints to island melanism are weak.

### (b) Dark dorsal plumage enhances camouflage of island birds

Our visual models revealed that island birds are generally more camouflaged against the island environment than mainland birds (figure 3). Island populations of scrub-jays, horned larks, house finches and orange-crowned warblers showed reduced chromatic and/or achromatic contrast with the island environment relative to mainland populations (figure 3; *p* ≤ 0.007 for all comparisons). For simplicity, we only report the results of violet-sensitive visual models in figure 3, but the results of ultraviolet-sensitive visual models are very similar (electronic supplementary material, figure S5). The differences in visual contrast between mainland and island birds largely occur above a commonly accepted threshold for visual discrimination (noise-weighted Euclidean distance = 2; [52]), suggesting that dark plumage reduces detection by predators. We found no differences in visual contrast with the island environment for rufous-crowned sparrow or spotted towhee populations (*p* ≥ 0.475 for all comparisons), consistent with weak mainland-island plumage divergence. We found no evidence that sex-related differences in feather reflectance or molt alter these findings (electronic supplementary material, figure S4 and table S12). Simulating visual contrast on the mainland largely eliminated the camouflage advantage of island birds (electronic supplementary material, figures S7 and S8), suggesting that dark plumage is adaptive only under island conditions.

### (c) Dark dorsal plumage reduces heating costs of island birds

**Figure 4.**
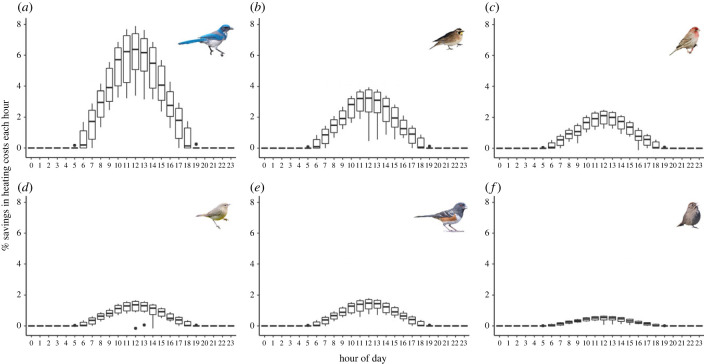
Island birds' dark plumage confers thermoregulatory benefits on Santa Cruz Island, especially at midday. The *y*-axis was calculated as: (net sensible heat flux of the mainland population − net sensible heat flux of the island population)/basal metabolic rate*100. Positive values indicate that birds with island plumage save energy associated with metabolic heat production compared to birds with mainland plumage. Simulations were run hourly over the course of a year, incorporating climatic conditions on Santa Cruz Island and assuming body masses typical of mainland populations. Combined with patterns of seasonal variation in thermoregulatory benefits (electronic supplementary material, figure S10), these results indicate that greater absorbance of solar irradiance by darker plumage drives the energetic benefits of island birds.

Consistent with the cool climate of Santa Cruz Island, birds incurred thermoregulatory heating costs (i.e. above basal metabolic costs) throughout much of the year (94% of daylight hours on average; minimum: 88.9% (scrub-jay), maximum: 97.9% (house finch)). We found that the darker plumage of island birds reduced their annual heating costs relative to mainland birds by an average of 1.62% (range: 0.316–4.49%) of their basal metabolic rate per hour (electronic supplementary material, figure S9). The magnitude of these energetic savings is comparable to the magnitude of basal metabolic rate variation affecting fitness in field studies of birds and mammals [53–55]. This suggests that the dark plumage of island birds, even of weakly divergent populations like rufous-crowned sparrows and spotted towhees, increases fitness through reductions in heating demands. We also found that hourly energetic savings for island birds were most pronounced at midday (average: 2.687%; range: 0.537–6.564%; figure 4) and tended to be greatest in May–July (electronic supplementary material, figure S10). This establishes the mechanism by which darker birds obtain an energetic benefit over lighter birds on Santa Cruz Island (i.e. greater solar radiation absorptance), because midday and May-July are when solar irradiance is greatest. Our simulation results are robust to heterothermy and mainland-island differences in body mass (electronic supplementary material, figure S11), both of which can influence thermoregulatory demands.

### (d) No evidence that elevated near-infrared reflectance is adaptive

Our thermoregulatory simulations found that even the darkest island birds rarely experience conditions that necessitate evaporative water loss on Santa Cruz Island. Horned larks and orange-crowned warblers incurred cooling costs during just 4% of daylight hours each year and scrub-jays incurred cooling costs during 11% of daylight hours each year. Although we found that the high near-infrared reflectance observed in these populations reduced evaporative water loss demands, these savings were three orders of magnitude lower than those considered physiologically relevant (maximum hourly water savings (% body mass): jay = 0.0051%; lark = 0.0059%; warbler = 0.0013%; [27]). We also note that manipulating near-infrared reflectance did not appreciably affect heating costs in our simulations (electronic supplementary material, figure S12). Collectively, we interpret these results as evidence that high near-infrared reflectance is not a compensatory adaptation in dark island bird populations.

### (e) The proximate basis of feather reflectance

Certain aspects of feather structure, especially the density of barbules, predict both visible and near-infrared reflectance [50]. Variation in barbule density could thus provide a mechanism driving the patterns of feather reflectance between island and mainland taxa (figure 2). However, we found no differences in barbule density of contour feathers between the darkest island individual and the lightest mainland individual of each taxon (electronic supplementary material, figure S13). Rather, we found that all California Channel Islands populations except rufous-crowned sparrows had lower illuminance of dorsal barbs than their mainland counterparts (electronic supplementary material, figure S13). We found no consistent mainland-island differences in ventral barb illuminance (electronic supplementary material, figure S13).

## 4. Discussion

### (a) Evolutionary trajectories of visible plumage divergence: possible causes of variation and implications for island melanism hypotheses

The species in our analyses last shared a common ancestor millions of years ago, yet the extent of convergence in visible dorsal plumage is comparable to or exceeds that of intraspecific phenotypic convergence in other well-known systems [56,57]. This points to natural selection as a probable driver of convergence, as alternative explanations (e.g. shared developmental/genetic constraints) are less likely when distantly related lineages converge on similar phenotypes [1,2]. Adaptive phenotypic plasticity, where feathers become darker in response to wet and/or cold conditions (e.g. [58]) could also contribute to convergence in this system. Despite the strong signal of convergence, we also found variation in the direction and magnitude of visible dorsal plumage trajectories. There are at least two possible explanations: divergence time and behavioural buffering. Although divergence time estimates are not available for all taxa, older island lineages (e.g. scrub-jay, horned lark, orange-crowned warbler; [30,34,36]) appear to have phenotypically diverged to a greater extent than younger island lineages (e.g. spotted towhee; [35]). Moreover, rufous-crowned sparrows and spotted towhees (the taxa showing the weakest plumage divergence) are behaviourally cryptic, rarely straying from dense patches of vegetation [59]. This behaviour might buffer these populations from strong predator- and climatic-mediated selection, dampening evolutionary responses of plumage (the ‘Bogert effect’; [60]). The only other California Channel Islands birds lacking island melanism (electronic supplementary material, figure S1) are another behaviourally cryptic sparrow (*Artemisiospiza belli*; [59]) and the islands' only hummingbird (*Selasphorus sasin*), which rarely experiences predation probably owing to its quick flight [61]. We thus speculate that divergence time and/or behavioural buffering explains variable plumage evolution in this system.

Unlike dorsal plumage, we found that mainland-island trajectories of ventral plumage evolution have not converged towards dark coloration. This finding mirrors those of comparative studies in birds and mammals over broader ecogeographic scales [18,62,63]. We suggest that the pattern of dorsal but not ventral darkening does not support other hypotheses for the evolution of island melanism. For example, some have proposed that darker plumage evolves on islands owing to relaxed intersexual selection [15,64] and/or relaxed selection for species recognition [13,15]. However, ventral feathers appear to play a more important role in social signalling than dorsal feathers [65–67]. It is thus unclear how relaxed intersexual selection or relaxed selection for species recognition would cause strong convergence towards darker dorsal but not ventral plumage. Another frequently invoked hypothesis posits that greater melanin deposition confers physical resistance to integument-degrading microbes, which are more abundant and keratinolytic in humid regions (the ‘microbe hypothesis’; [68]). However, most keratinolytic bacteria occur in soil which ventral surfaces are often in contact with, resulting in greater microbial prevalence on ventral than dorsal surfaces [69]. The microbe hypothesis thus predicts strong and convergent evolutionary responses towards dark ventral coloration, the opposite of our results. Furthermore, most diurnal mammals endemic to the California Channel Islands have evolved darker pelage than their mainland relatives, but most nocturnal mammals have not [70,71]. It is not clear how the microbe hypothesis could account for this pattern or the differences in ventral and dorsal plumage evolution we describe, though we acknowledge that relatively little work on this topic exists [72]. Conversely, these patterns are consistent with hypotheses proposing that camouflage from diurnal visual predators and/or thermoregulatory benefits from elevated solar absorptance are the major fitness benefits of dark plumage in the California Channel Islands.

### (b) Visual predation as an agent of selection favouring dark island plumage

Pitelka [23] and Johnson [14] hypothesized that the dark dorsal plumage of California Channel Islands birds is an adaptation to enhance camouflage from diurnal raptors. Similar hypotheses have been proposed to explain broader climate-colour associations in other taxa [18], and there is evidence that island melanism is most common on islands with many diurnal raptors [16]. Our violet- and ultraviolet-sensitive avian visual models, which approximate the visual physiology of diurnal predatory birds [49], support these hypotheses. The dorsal plumage of all island populations except rufous-crowned sparrows and spotted towhees reduces chromatic and/or achromatic contrast with the island environment relative to mainland plumage. The reductions in visual contrast conferred by dark plumage occur above the visual discrimination threshold assumed for most birds [52]. Furthermore, both chromatic and achromatic contrast are important for diurnal raptor foraging [73], indicating that the dark dorsal plumage of island birds reduces their conspicuousness to predators ([74]; but see [75]). The relationship between plumage phenotype and camouflage was less clear under mainland environmental conditions (i.e. lighter soils, brighter skies). Specifically, there was no clear pattern of reduced chromatic or achromatic contrast for island or mainland plumage phenotypes. One explanation is that dark colour enhances camouflage in low light environments (such as the California Channel Islands), but brightness alone does not affect conspicuousness to predators in environments with high light intensity [76]. Light colours may still be favoured in light environments if they enhance other effects that reduce conspicuousness such as pattern-matching [20]. Collectively, these results support the hypothesis that predation plays a role in shaping colour-environment associations, though its effect may be more nuanced than generally assumed.

### (c) Temperature, thermoregulation and endotherm coloration

While many studies have explored mechanistic links between temperature and ecogeographic patterns of ectotherm coloration [9], comparatively little work has been done on endotherms. Early work described a seemingly maladaptive correlation between dark endotherm coloration and warm temperatures [10]. This pattern, combined with the general decoupling of body and ambient temperatures in endotherms, suggests that temperature may not exert strong selection on endotherm coloration. However, more rigorous comparative studies that account for humidity have found that dark endotherm coloration is more often correlated with cold temperatures [7,8]. There is also a growing realization that endotherms' physiological relationship with temperature is more complex than historically assumed (e.g. [77]). This coincides with the emergence of studies that have found support for thermoregulatory explanations of colour-climatic associations in endotherms [6,20,78].

Our heat flux simulations add to this emerging literature by providing evidence that dark plumage reduces thermoregulatory demands of birds in a cool, thermally challenging environment. We modelled conditions on Santa Cruz Island, which is the warmest and least windy of the California Channel Islands [21]. Consequently, our estimates of energetic savings associated with dark plumage probably represent the lower bound experienced by California Channel Islands birds. Still, we found that island populations with even marginally darker plumage than their mainland counterparts experience reductions in energetic demands that could increase fitness [53–55]. Dark birds experience energetic savings throughout the year, but we found that savings tended to be greatest during the breeding season, when the energetic demands of adults are at their highest [79]. Small yet significant energetic savings at this time could reduce the likelihood of nest abandonment during inclement weather [80] or the need to engage in risky foraging behaviour that increases predation risk [81]. While more work is needed to explore the effects on fitness, our results suggest that dark plumage may be part of a suite of adaptations to cold temperature in California Channel Islands songbirds [82]. More generally, we suspect that temperature is an important yet understudied selective agent shaping ecogeographic patterns of colour variation in endotherms.

### (d) Evolutionary dynamics of near-infrared feather reflectance

Most solar radiation occurs in near-infrared wavelengths that are invisible to animals [83]. Therefore, altered near-infrared reflectance should allow animals to control heat flux without compromising vital functions of visible light reflectance (e.g. camouflage, sexual signalling; [83]). Recent work in a variety of taxa including birds supports this possibility: organisms in hot environments have generally evolved high near-infrared reflectance, presumably to reduce heat gain [27,84]. Interestingly, we found that high near-infrared reflectance evolved in island populations that also evolved the darkest visible plumage. We thus hypothesized that high near-infrared reflectance in these populations is a compensatory adaptation that reduces heat gain associated with dark visible plumage. However, the savings in evaporative water loss from high near-infrared reflectance in our simulations are substantially lower than the minimum values considered physiologically important in comparable studies [27].

If elevated near-infrared reflectance is not adaptive on the California Channel Islands, why has it evolved at least three times in distantly related taxa? Selection on near-infrared reflectance is probably weak because our simulations indicate it does not affect thermoregulation, nor is it visually detected by predators or mates. Weak selection and small population sizes in California Channel Islands birds (e.g. [30]) are conditions where genetic drift could drive convergent evolution [3]. Another possibility is that high near-infrared reflectance is a non-adaptive byproduct of dark visible plumage (a ‘spandrel’, *sensu* Gould & Lewontin [85]). Because barbule density is a strong predictor of visible and near-infrared reflectance across species [50], we predicted that barbule density differs between island and mainland birds. We found no evidence for this based on comparisons of feathers from the darkest island and lightest mainland individuals from each taxon, though additional comparisons are needed to draw robust conclusions about mainland-island differences in feather microstructure. Our comparisons of feathers did reveal that dorsal barb illuminance is lower in California Channel Islands birds than mainland birds. Variation in melanin deposition is thought to underlie ecogeographic variation in plumage darkness [10] and may explain our visible reflectance and barb illuminance results. However, variation in melanin appears to have no effect on variation in near-infrared reflectance [86], indicating that other feather components underlie this pattern, such as feather density or the chemical composition of feathers. Regardless of the proximal causes, our results suggest that caution is warranted when ascribing adaptive significance to near-infrared reflectance without evaluating its effects on performance.

## 5. Conclusion

Testing the adaptive significance of colour-environmental associations is notoriously difficult, resulting in numerous hypotheses that have remained largely untested for over a century [87]. By combining multivariate trajectory analyses with visual and thermoregulatory modelling, our study provides evidence that birds with dark plumage have an energetic and camouflage advantage over lighter birds on Santa Cruz Island. We also found a previously undescribed pattern of elevated near-infrared feather reflectance in the most divergent island populations, but no evidence that it is adaptive. Although our study focused on a classic case of convergent local adaptation over a relatively small spatial and environmental scale, the adaptive dynamics we describe may underlie broader ecogeographic patterns of organismal coloration. More generally, the techniques we used here may represent a powerful approach for evaluating the importance of adaptive and non-adaptive mechanisms underlying convergent evolutionary responses.

## Data Availability

All data and code associated with this manuscript are available at Dryad (https://doi.org/10.5061/dryad.4f4qrfjjf) [28]. Supplementary material is available online [88].

## References

[1] Losos JB. 2011 Convergence, adaptation, and constraint. Evolution **65**, 1827-1840. (10.1111/j.1558-5646.2011.01289.x) 21729041

[2] Bolnick DI, Barrett RDH, Oke KB, Rennison DJ, Stuart YE. 2018 (Non)parallel evolution. Annu. Rev. Ecol. Evol. Syst. **49**, 303-330. (10.1146/annurev-ecolsys-110617-062240)

[3] Lerio AM, Rose MR, Lauder GV. 1994 What does the comparative method reveal about adaptation? Am. Nat. **143**, 381-402. (10.1086/285609)

[4] Naumann B, Schweiger S, Hammel JU, Müller H. 2021 Parallel evolution of direct development in frogs – skin and thyroid gland development in African squeaker frogs (Anura: Arthroleptidae: *Arthroleptis*). Dev. Dyn. **250**, 584-600. (10.1002/dvdy.275) 33354814

[5] Delhey K. 2018 Darker where cold and wet: Australian birds follow their own version of Gloger's rule. Ecography **41**, 673-683. (10.1111/ecog.03040)

[6] Galván I, Rodríguez-Martínez S, Carrascal LM. 2018 Dark pigmentation limits thermal niche position in birds. Funct. Ecol. **32**, 1531-1540. (10.1111/1365-2435.13094)

[7] Dalrymple RL, Flores-Moreno H, Kemp DJ, White TE, Laffan SW, Hemmings FA, Hitchcock TD, Moles AT. 2018 Abiotic and biotic predictors of macroecological patterns in bird and butterfly coloration. Ecol. Monogr. **88**, 204-224. (10.1002/ecm.1287)

[8] Delhey K, Dale J, Valcu M, Kempenaers B. 2019 Reconciling ecogeographical rules: rainfall and temperature predict global colour variation in the largest bird radiation. Ecol. Lett. **22**, 726-736. (10.1111/ele.13233) 30779293

[9] Clusella-Trullas S, Terblanche JS, Blackburn TM, Chown SL. 2008 Testing the thermal melanism hypothesis: a macrophysiological approach. Funct. Ecol. **22**, 232-238. (10.1111/j.1365-2435.2007.01377.x)

[10] Delhey K. 2019 A review of Gloger's rule, an ecogeographical rule of colour: definitions, interpretations and evidence. Biol. Rev. **94**, 1294-1316. (10.1111/brv.12503) 30892802

[11] Krah F et al. 2019 European mushroom assemblages are darker in cold climates. Nat. Commun. **10**, 2890. (10.1038/s41467-019-10767-z) 31253790 PMC6599080

[12] Martínez-Freiría F, Toyama KS, Freitas I, Kaliontzopoulou A. 2020 Thermal melanism explains macroevolutionary variation of dorsal pigmentation in Eurasian vipers. Sci. Rep. **10**, 16122. (10.1038/s41598-020-72871-1) 32999337 PMC7528074

[13] Grant PR. 1965 Plumage and the evolution of birds on islands. Syst. Zool. **14**, 47-52. (10.2307/2411902)

[14] Johnson NK. 1972 Origin and differentiation of the avifauna of the Channel Islands, California. Condor **74**, 295-315. (10.2307/1366591)

[15] Doutrelant C, Paquet M, Renoult JP, Grégoire A, Crochet P, Covas R. 2016 Worldwide patterns of bird colouration on islands. Ecol. Lett. **19**, 537-545. (10.1111/ele.12588) 26932367

[16] Bliard L, Paquet M, Robert A, Dufour P, Renoult JP, Grégoire A, Crochet P, Covas R, Doutrelant C. 2020 Examining the link between relaxed predation and bird coloration on islands. Biol. Lett. **16**, 20200002. (10.1098/rsbl.2020.0002) 32315593 PMC7211456

[17] Weigelt P, Jetz W, Kreft H. 2013 Bioclimatic and physical characterization of the world's islands. Proc. Natl Acad. Sci. USA **110**, 15 307-15 312. (10.1073/pnas.1306309110) PMC3780862 24003123

[18] Zink RM, Remsen JV. 1986 Evolutionary processes and patterns of geographic variation in birds. In Current ornithology (ed. RF Johnston), pp. 1-69. New York, NY: Plenum Press.

[19] Rogalla S, Shawkey MD, D'Alba L. 2022 Thermal effects of plumage coloration. Ibis **164**, 933-948. (10.1111/ibi.13100)

[20] Mason NA, Riddell EA, Romero FG, Cicero C, Bowie RCK. 2023 Plumage balances camouflage and thermoregulation in horned larks (*Eremophila alpestris*). Am. Nat. **201**, E23-E40. (10.1086/722560) 36724466

[21] Schoenherr AA, Feldmeth CR, Emerson MJ. 2003 Natural history of the islands of California. Oakland, CA: University of California Press.

[22] Dewitz J. 2021 U.S. Geological Survey, National Land Cover Database Products (version 2.0). See https://www.usgs.gov/centers.eros/science/national-land-cover-database.

[23] Pitelka FA. 1951 Speciation and ecological distribution of American jays of the genus *Aphelocoma*. Univ. of California Publ. Zool. **50**, 195-464.

[24] Dunne P, Karlson KT. 2017 Birds of prey: hawks, eagles, falcons, and vultures. New York, NY: HarperCollins.

[25] Vignieri SN, Larson JG, Hoekstra HE. 2010 The selective advantage of crypsis in mice. Evolution **64**, 2153-2158. 20163447 10.1111/j.1558-5646.2010.00976.x

[26] Boratyński Z, Brito JC, Campos JC, Cunha JL, Granjon L, Mappes T, Ndiaye A, Rzebik-Kowalska B, Serén N. 2017 Repeated evolution of camouflage in speciose desert rodents. Sci. Rep. **7**, 3522. (10.1038/s41598-017-03444-y) 28615685 PMC5471182

[27] Medina I, Newton E, Kearney MR, Mulder RA, Porter WP, Stuart-Fox D. 2018 Reflection of near-infrared light confers thermal protection in birds. Nat. Commun. **9**, 3610. (10.1038/s41467-018-05898-8) 30190466 PMC6127310

[28] Porter CK, Romero FG, Adams DC, Bowie RCK, Riddell EA. 2023 Data from: Adaptive and non-adaptive convergent evolution in feather reflectance of California Channel Islands songbirds. Dryad Digital Repository. (10.5061/dryad.4f4qrfjjf) PMC10646447 37964520

[29] Riddell EA, Iknayan KJ, Wolf BO, Beissinger SR. 2019 Cooling requirements fueled the collapse of a desert bird community from climate change. Proc. Natl Acad. Sci. USA **116**, 21 609-21 615. (10.1073/pnas.1908791116) PMC6815107 31570585

[30] Delaney KS, Zafar S, Wayne RK. 2008 Genetic divergence and differentiation within the western scrub-jay (*Aphelocoma californica*). Auk **125**, 839-849. (10.1525/auk.2008.07088)

[31] Caballero IC, Ashley MV. 2011 Genetic analysis of the endemic island loggerhead shrike, *Lanius ludovicianus anthonyi*. Conserv. Genet. **12**, 1485-1493. (10.1007/s10592-011-0247-4)

[32] Wilson AG, Arcese P, Chan YL, Patten MA. 2011 Micro-spatial genetic structure in song sparrows (*Melospiza melodia*). Conserv. Genet. **12**, 213-222. (10.1007/s10592-010-0134-4)

[33] Wilson AG, Chan YL, Taylor SS, Arcese P. 2015 Genetic divergence of an avian endemic on the Californian Channel Islands. PLoS ONE **10**, e0134471. 26308717 10.1371/journal.pone.0134471 PMC4550415

[34] Mason NA, Title PO, Cicero C, Burns KJ, Bowie RCK. 2014 Genetic variation among western populations of the horned lark (*Eremophila alpestris*) indicates recent colonization of the Channel Islands off southern California, mainland-bound dispersal, and postglacial range shifts. Auk **131**, 162-174. (10.1642/AUK-13-181.1)

[35] Walsh SE. 2015 Genetic and phenotypic divergence of the spotted towhee (*Pipilo maculatus*) on the California Channel Islands. Masters thesis, San Diego State University, San Diego, CA, USA.

[36] Hanna ZR, Cicero C, Bowie RCK. 2019 Molecular evidence that the Channel Islands populations of the orange-crowned warbler (*Oreothlypis celata*; Aves: Passeriformes: Parulidae) represent a distinct evolutionary lineage. PeerJ **7**, e7388. (10.7717/peerj.7388) 31404458 PMC6688592

[37] Gates DM. 1980 Biophysical ecology. Mineola, NY: Dover Publications.

[38] Collyer ML, Adams DC. 2018 RRPP: An R package for fitting linear models to high-dimensional data using residual randomization. Methods Ecol. Evol. **9**, 1772-1779. (10.1111/2041-210X.13029)

[39] Adams DC, Nistri A. 2010 Ontogenetic convergence and evolution of foot morphology in European cave salamanders (Family: Plethodontidae). BMC Evol. Biol. **10**, 216. (10.1186/1471-2148-10-216) 20637087 PMC2927916

[40] Gamboa MP. 2021 Evolutionary underpinnings of microgeographic adaptation in song sparrows distributed along a steep climate gradient. PhD dissertation, Colorado State University, Fort Collins, CO, USA.

[41] Morrow CB, Morgan Ernest SK, Kerkhoff AJ. 2021 Macroevolution of dimensionless life-history metrics in tetrapods. Proc. R. Soc. B **288**, 20210200. (10.1098/rspb.2021.0200) PMC8079996 33906402

[42] Fritz SA, Purvis A. 2010 Selectivity in mammalian extinction risk and threat types: a new measure of phylogenetic signal strength in binary traits. Conserv. Biol. **24**, 1042-1051. (10.1111/j.1523-1739.2010.01455.x) 20184650

[43] Maia R, Gruson H, Endler JA, White TE. 2019 PAVO 2: new tools for the spectral and spatial analysis of colour in R. Methods Ecol. Evol. **10**, 1097-1107. (10.1111/2041-210X.13174)

[44] Vorobyev M, Osorio D. 1998 Receptor noise as a determinant of colour thresholds. Proc. R. Soc. B **265**, 351-358. (10.1098/rspb.1998.0302) PMC1688899 9523436

[45] Beaudette DE, Roudier P, O'Green AT. 2013 Algorithms for quantitative pedology: a toolkit for soil scientists. Comput. Geosci. **52**, 258-268. (10.1016/j.cageo.2012.10.020)

[46] Burns SA. 2019 Numerical methods for smoothest reflectance reconstruction. Color Res. Appl. **45**, 8-21. (10.1002/col.22437)

[47] Kokaly RF, Despain DG, Clark RN, Livo KE. 2003 Mapping vegetation in Yellowstone National Park using spectral feature analysis of AVIRIS data. Remote Sens. Environ. **84**, 437-456. (10.1016/S0034-4257(02)00133-5)

[48] Collins PW, Jones HL. 2015 A checklist of birds of the California Channel Islands. Santa Barbara, CA: Santa Barbara Museum of Natural History.

[49] Lind O, Mitkus M, Olsson P, Kelber A. 2013 Ultraviolet sensitivity and colour vision in raptor foraging. J. Exp. Biol. **216**, 1819-1826. (10.1242/jeb.082834) 23785106

[50] Stuart-Fox D, Newton E, Mulder RA, D'Alba L, Shawkey MD, Igic B. 2018 The microstructure of white feathers predicts their visible and near-infrared reflectance properties. PLoS ONE **13**, e0199129. (10.1371/journal.pone.0199129) 29975724 PMC6033395

[51] Collyer ML, Adams DC. 2013 Phenotypic trajectory analysis: comparison of shape change patterns in evolution and ecology. Hystrix It. J. Mamm. **24**, 75-83.

[52] Endler JA, Mielke PW. 2005 Comparing color patterns as birds see them. Biol. J. Linn. Soc. **86**, 405-431. (10.1111/j.1095-8312.2005.00540.x)

[53] Jackson DM, Trayhurn P, Speakman JR. 2001 Associations between energetics and over-winter survival in the short-tailed field vole *Microtus agrestis*. J. Anim. Ecol. **70**, 633-640. (10.1046/j.1365-2656.2001.00518.x)

[54] Larivée ML, Boutin S, Speakman JR, McAdam AG, Humphries MM. 2010 Associations between over-winter survival and resting metabolic rate in juvenile North American red squirrels. Funct. Ecol. **24**, 597-607. (10.1111/j.1365-2435.2009.01680.x)

[55] Rønning B, Broggi J, Bech C, Moe B, Ringsby TH, Pärn H, Hagen IJ, Sæther B, Jensen H. 2015 Is basal metabolic rate associated with recruit production and survival in free-living house sparrows? Funct. Ecol. **30**, 1140-1148. (10.1111/1365-2435.12597)

[56] Stuart YE et al. 2017 Contrasting effects of environment and genetics generate a continuum of parallel evolution. Nat. Ecol. Evol. **1**, 158. (10.1038/s41559-017-0158) 28812631

[57] Weber AAT, Rajkov J, Smailus K, Egger B, Salzburger W. 2021 Speciation dynamics and extent of parallel evolution along a lake-stream environmental contrast in African cichlid fishes. Sci. Adv. **7**, eabg5391. (10.1126/sciadv.abg5391) 34731007 PMC8565912

[58] López-Rull I, Salaberría C, Fargallo JA. 2023 Plastic plumage colouration in response to experimental humidity supports Gloger's rule. Sci. Rep. **13**, 858. (10.1038/s41598-023-28090-5) 36646811 PMC9842646

[59] Billerman SM, Keeney BK, Rodewald PG, Schulenberg TS. 2022 Birds of the world. Ithaca, NY: Cornell Laboratory of Ornithology.

[60] Muñoz MM. 2022 The Bogert effect, a factor in evolution. Evolution **76**, 49-66. (10.1111/evo.14388) 34676550

[61] Miller RS, Gass CL. 1985 Survivorship in hummingbirds: is predation important? Auk **102**, 175-178. (10.2307/4086840)

[62] Friedman NR, Remeš V. 2017 Ecogeographical gradients in plumage coloration among Australasian songbird clades. Global Ecol. Biogeogr. **26**, 261-274. (10.1111/geb.12522)

[63] Marcondes RS, Nations JA, Seeholzer GF, Brumfield RT. 2021 Rethinking Gloger's rule: climate, light environments, and color in a large family of tropical birds (Furnariidae). Am. Nat. **197**, 592-606. (10.1086/713386) 33908827

[64] Griffith SC. 2000 High fidelity on islands: a comparative study of extrapair paternity in passerine birds. Behav. Ecol. **11**, 265-273. (10.1093/beheco/11.3.265)

[65] Doucet S, Mennill DJ, Hill GE. 2007 The evolution of signal design in manakin plumage ornaments. Am. Nat. **169**, 62-80. (10.1086/510162) 29517930

[66] Seddon N et al. 2013 Sexual selection accelerates signal evolution during speciation in birds. Proc. R. Soc. B **280**, 20131065. (10.1098/rspb.2013.1065) PMC3730587 23864596

[67] Friedman NR, Remeš V. 2015 Rapid evolution of elaborate male coloration is driven by visual system in Australian fairy-wrens (Maluridae). J. Evol. Biol. **28**, 2125-2135. (10.1111/jeb.12737) 26299546

[68] Burtt Jr EH, Ichida JM. 2004 Gloger's Rule, Feather-degrading bacteria, and color variation among song sparrows. Condor **106**, 681-686. (10.1093/condor/106.3.681)

[69] Burtt Jr EH, Ichida JM. 1999 Occurrence of feather-degrading bacilli in the plumage of birds. Auk **116**, 364-372. (10.2307/4089371)

[70] von Bloeker Jr JC. 1965 Land mammals of the southern California Islands. In 1st symposium on the biology of the California islands, pp. 245-263. Washington, DC: National Park Service.

[71] Collins PW. 1982 Origin and differentiation of the island fox: a study of evolution in insular populations. Masters thesis, University of California Santa Barbara, Santa Barbara, CA, USA.

[72] Kent CM, Burtt Jr EH. 2016 Feather-degrading bacilli in the plumage of wild birds: prevalence and relation to feather wear. Auk **133**, 583-592. (10.1642/AUK-16-39.1)

[73] Moore BA, Montiani-Ferreira F. 2022 Ophthalmology of Accipitrimorphae, Strigidae, and Falconidae: hawks, eagles, vultures, owls, falcons, and relatives. In Wild and exotic animal ophthalmology (eds F Montiani-Ferreira, BA Moore, G Ben-Shlomo), pp. 429-504. New York, NY: Springer Nature.

[74] Ruiz-Rodríguez M et al. 2013 Does avian conspicuous colouration increase or reduce predation risk? Oecologia **173**, 83-93. (10.1007/s00442-013-2599-6) 23386048

[75] Cain KE et al. 2019 Conspicuous plumage does not increase predation risk: a continent-wide test using model songbirds. Am. Nat. **193**, 359-372. (10.1086/701632) 30794446

[76] Cheng W, Xing S, Chen Y, Lin R, Bonebrake TC, Nakamura A. 2018 Dark butterflies camouflaged from predation in dark tropical forest understories. Ecol. Entomol. **43**, 304-309. (10.1111/een.12499)

[77] Levesque DL, Marshall KE. 2021 Do endotherms have thermal performance curves? J. Exp. Biol. **224**, jeb141309. (10.1242/jeb.141309) 33536290

[78] Wacker CB, McAllan BM, Körtner G, Geiser F. 2016 The functional requirements of mammalian hair: a compromise between crypsis and thermoregulation? Sci. Nat. **103**, 53. (10.1007/s00114-016-1376-x) 27287044

[79] Weathers WW, Sullivan KA. 1993 Seasonal patterns of time and energy allocation by birds. Physiol. Zool. **66**, 511-536. (10.1086/physzool.66.4.30163806)

[80] Merilä J, Wiggins DA. 1997 Mass loss in breeding blue tits: the role of energetic stress. J. Anim. Ecol. **66**, 452-460. (10.2307/5940)

[81] Powolny T, Bretagnolle V, Aguilar A, Eraud C. 2014 Sex-related differences in the trade-off between foraging and vigilance in a granivorous forager. PLoS ONE **9**, e101598. (10.1371/journal.pone.0101598) 24984028 PMC4077796

[82] Greenberg R, Danner RM. 2012 The influence of the California marine layer on bill size in a generalist songbird. Evolution **66**, 3825-3835. (10.1111/j.1558-5646.2012.01726.x) 23206140

[83] Stuart-Fox D, Newton E, Clusella-Trullas S. 2017 Thermal consequences of colour and near-infrared reflectance. Phil. Trans. R. Soc. B **372**, 20160345. (10.1098/rstb.2016.0345) 28533462 PMC5444066

[84] Kang C, Im S, Lee WY, Choi Y, Stuart-Fox D, Huertas B. 2021 Climate predicts both visible and near-infrared reflectance in butterflies. Ecol. Lett. **24**, 1869-1879. (10.1111/ele.13821) 34174001

[85] Gould SJ, Lewontin RC. 1979 The spandrels of San Marco and the Panglossian paradigm: a critique of the adaptationist programme. Proc. R. Soc. B **205**, 581-598. 42062 10.1098/rspb.1979.0086

[86] Li W, Patil A, Zhou W, Xiao M, Shawkey MD, Gianneschi NC, Dhinojwala A. 2020 Characterization of broadband complex refractive index of synthetic melanin coatings and their changes after ultraviolet irradiation. Appl. Phys. Lett. **117**, 203701. (10.1063/5.0024229)

[87] Caro T. 2005 The adaptive significance of coloration in mammals. BioScience **55**, 125-136. (10.1641/0006-3568(2005)055[0125:TASOCI]2.0.CO;2)

[88] Porter CK, Romero FG, Adams DC, Bowie RCK, Riddell EA. 2023 Adaptive and non-adaptive convergent evolution in feather reflectance of California Channel Islands songbirds. Figshare. (10.6084/m9.figshare.c.6919586) PMC10646447 37964520

